# Male breast cancer in the prophylactics programme by the Ministry of Health of Poland

**DOI:** 10.1186/1897-4287-10-S4-A12

**Published:** 2012-12-10

**Authors:** Artur Barczyk

**Affiliations:** 1 Department of Medical Genetics, Medical Academy, Łódz, Poland

## 

The propyhlaxy programme for the families with high hereditary risk of malignant cancers organized and guided by the Ministry of Health of Poland. Our data are based on the activity of the ZOZ “SALVE”, one of three units operating in the Lodz district in Poland. Over 600 questionnaires produced by patients with problems of in-family cases of cancer (including ovary cancers and/or breast cancer) were collected between mid-2010 to the end of the 2011. Four cases of male breast cancer were recorded and screened across the clinically recorded family data for these patients. It appeared that these cases could not be fully explained in accordance with the current concept of the family cases of cancers. These discrepances could be either related to the faulty selection criteria or to the highly differentiated male population suffering the breast cancer. The diagnostic potential within the “Module 1: early detection of malignant cancers in hereditary high-risk families with ovary cancer and breast cancer“ is discussed and evaluated in practical terms including regulatory/ legislation aspects. Supported by the Ministry of Health of Poland.

